# An arcane case report of primary intramuscular hydatid cyst of thigh

**DOI:** 10.1016/j.ijscr.2021.01.089

**Published:** 2021-01-27

**Authors:** Vaibhav Lakhanpal, Mohit Badgurjar, Pankaj Saxena

**Affiliations:** Department of General Surgery, Geetanjali Medical College and Hospital, Udaipur, India

**Keywords:** HD, Hydatid Disease, USG, Ultrasonography, MRI, Magnetic Resonance Imaging, CECT, Contrast Enhanced Computed Tomography, CT, Computed Tomography, Case report, Hydatid disease, Intramuscular hydatidosis, Pericystectomy

## Abstract

**Introduction:**

Hydatid diseases are a cystic parasitic infestation caused by Echinococci which are endemic to parts of India and commonly occur in agricultural workers. Primary intramuscular hydatidosis is a rare manifestation of hydatid disease.

**Presentation of case:**

Our case report describes a rare case of primary intramuscular hydatidosis in an agricultural worker with no involvement of any other viscera. This was diagnosed with clinical evaluation, radiological modalities and managed surgically.

**Discussion:**

Hydatid disease is endemic in parts of India. Primary intramuscular hydatidosis is uncommon because the lactic acid in the muscle and muscle contractility hinders the development of cysts making it improbable diagnosis on first presentation. Hydatid cyst demonstrates a wide variety of imaging features, which can vary according to growth stage, associated complications and affected tissue. The treatment of choice is complete surgical excision of the cyst along with thorough irrigation of the surrounding soft tissues combined with the use of systemic antiparasitic drugs after surgery.

**Conclusion:**

In a patient of an agricultural background of cattle rearing living in an endemic region and presenting with a swelling in the musculoskeletal system, a suspicion of hydatidosis should always be kept in the clinicians mind, so that it can be diagnosed using a multimodal approach and managed properly in a timely manner.

## Highlights

• Intramuscular hydatidosis is an extremely rare manifestation of hydatid disease which is endemic in India.
• Patient presented with a large swelling over the left thigh and was diagnosed in a timely manner using a multimodal approach.
• The rare location of the disease is confounding and required thorough and meticulous evaluation.
• Upon surgical exploration, the presence of multiple cysts and daughter cysts proved to be challenging but complete pericystectomy was achieved.

## 1 Introduction

Hydatid diseases (HDs) are an endemic cystic parasitic infestation caused by Echinococci which are the cestode of the Taeniidae family. These are commonly encountered in farm workers dealing with agriculture and livestock [1]. The organisms have both definitive hosts (dogs) and intermediate hosts (cattle, sheep, humans) [2]. In humans, the most frequently affected organs are liver, lung and brain [3]. Primary hydatid cyst located in the musculoskeletal system is uncommon even in endemic regions [4]. The patient could present with a slow growing large swelling in the extremities, which on clinical examination may give the appearance of a soft tissue tumour such as a sarcoma, liposarcoma or a lipoma. This case report is in line with SCARE criteria [5].

## 2 Case study

**Fig. 1:**
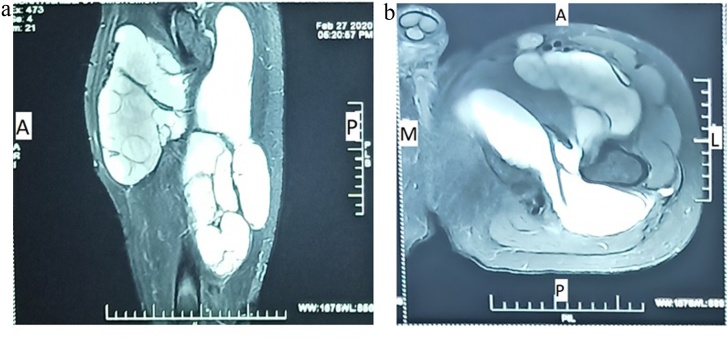
MRI showing intramuscular hydatid cyst with multiple daughter cysts in the left thigh (a) Coronal section (b) Axial section.

A 52-year-old male working as a cattle rearer in a farm in rural India, presented with complaints of swelling over left thigh region. Disease onset was 2 years ago when patient first noticed a small swelling over left thigh. Swelling was insidious in onset, spontaneous in origin and gradually progressed to a size of 18 × 12 cm. There was no period of rapid increase or regression in size of swelling. There was no history of pain over swelling. There was no history of trauma or fever. No history of any comorbidities. General examination of the patient showed no irregularities. Physical examination showed a firm, non-tender mass of the upper two third of the left thigh. Overlying skin was normal. No other visible or palpable swelling was present in the rest of the body. All routine blood investigations were normal. Ultrasonography (USG) revealed large multi-loculated complex cystic mass measuring 31 × 29 × 19 cm (volume 236 mL) in the anterior and medial aspect of the upper two third of left thigh predominantly in subcutaneous plane. Magnetic Resonance Imaging (MRI) revealed well encapsulated cystic lesions comprising of multiple daughter cysts of varying sizes within them, involving the anterior and posterior compartments of left thigh (predominantly involving the vastus medialis and adductor muscles) measuring approx. 17.2 cm (SI) × 10.6 cm (TR) × 8.7 cm (AP) and 25 cm (SI) × 17 cm (TR) × 7 cm (AP) respectively. The lesions showed mild peripheral enhancement on post contrast study and were seen causing compression over the adjacent muscles and anterior neurovascular bundle. No skeletal involvement was seen (Fig. 1).

Chest X-ray and Contrast Enhanced Computed Tomography (CECT) abdomen pelvis and chest did not reveal any other organ involvement. On the basis of these findings a diagnosis of intramuscular hydatid cyst of the left thigh was established and surgical excision was planned. Post-operative histopathological examination confirmed our diagnosis of intramuscular hydatid cyst.

## 3 Intervention

**Fig. 2:**
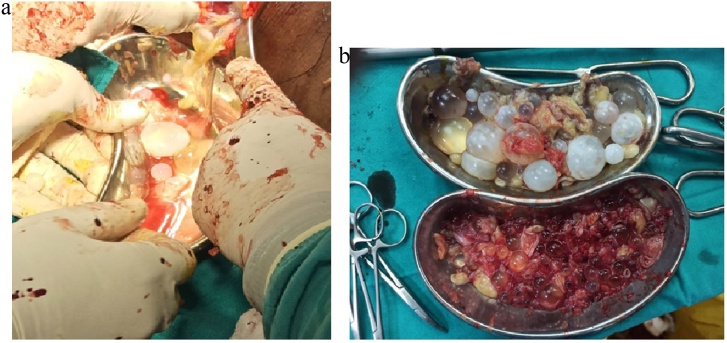
(a) removal of daughter cysts following the opening of a large hydatid cyst (b) multiple evacuated daughter cysts.

Under spinal anesthesia, 2 incisions were given over left thigh. Vertical incision anterolaterally (9 cm) and oblique incision medially (13 cm). 3 large cysts were located in the anterolateral, anteromedial and posteromedial compartments. The anteromedial and posteromedial cysts were found to be communicating. Posteromedial cyst extended upto gluteal region. After securing the wound with sponge soaked in hypertonic saline (3%), the cyst was opened up and upto 2 L of dirty putrefied material along with daughter cysts were removed. Pericystectomy was done for all cysts along with evacuation of daughter cysts. Thorough wash was given with 10 % povidone iodine. Two abdominal drains (28Fr) were inserted in the anteromedial and posteromedial compartments and the wound was closed primarily. Post-operative period was uneventful. Both drains were removed on the second day. Patient was discharged on the third day (Fig. 2).

## 4 Follow up

On discharge, patient was advised albendazole therapy for one month. Follow up was advised at 15 days, 1 month, and 3 months. Patient had no local or systemic complications. There were no clinical signs of recurrence. USG was repeated on each follow up and showed no residual cysts or recurrence.

## 5 Discussion

Hydatid disease is an endemic zoonosis caused by Echinococcus granulosus prevalent mainly in countries where sheep raising is practiced; however, due to increased travel and tourism around the world, it can be found anywhere, even in developed countries. In India, hydatid disease is commonly seen in the southern states of Andhra Pradesh and Tamil Nadu [6].

Hydatid disease can be primary or secondary. Primary hydatid disease is spread by the ingestion of the ova and may develop in almost any organ. Liver and lungs are the most frequently affected organs. In secondary hydatid disease, larval tissue proliferates after spreading from the primary site. However, there is a primary site of hydatid disease situated in the liver, lung, or spleen [7].

Hydatid cyst located primarily in the intramuscular tissue is an extremely rare manifestation of the hydatid disease. Patients usually present with a painless, slow growing mass with normal overlying skin [7]. Primary intramuscular hydatidosis is uncommon because the lactic acid in the muscle and muscle contractility hinders the development of cysts. Parasitic cysts have a tendency to grow around the muscles of the neck, trunk and roots of the limbs where there is less muscular activity and more vascularization. These cysts develop slowly and over time progress to act as space occupying lesions which subsequently cause pressure related effects on the tissues surrounding the lesions [8].

The pathogenesis of muscular localization remains a matter of debate. Some authors claim that it is because of direct implantation through a wound, for instance, through the bite of a dog. However, most authors believe that the embryo reaches the muscles via systemic circulation after leaving the intestine and passing through the liver and the lungs which act as filters [9]. Soft tissue tumors, traumatic and developmental lesions are some important differential diagnoses. If the list of differential diagnoses includes hydatid cyst, incisional biopsy and marginal excision are contraindicated in order to limit the risk of disease dissemination and anaphylaxis. Cystic fluid contains large amounts of foreign protein and is extremely toxic to the host [10].

Other possible primary locations of the disease must be ruled out by careful clinical and radiological examination of the patient. The Casoni skin test and various serological tests are available for the diagnosis of hydatidosis. They may help to distinguish hydatid cyst from other non-parasitic cysts and abscesses but can give negative results in case of encapsulation of the lesion. Thus, a negative test does not rule out the possibility of hydatid cyst [11].

USG, CT, and MRI are used to describe the location and features of the cyst. Hydatid cyst can demonstrate a wide variety of imaging features, which can vary according to growth stage, associated complications, and affected tissue. The radiologic findings can range from purely cystic lesions to a completely solid appearance [12].

USG is a good diagnostic tool and has a high diagnostic accuracy. USG is particularly useful for the detection of cystic membranes, septa and hydatid sand, floating membranes, daughter cysts and vesicles. CT is a useful modality to demonstrate cyst wall calcification, the internal cystic structure posterior to calcification and cyst infection [13]. However, endovesicular daughter cysts that are commonly seen in radio imaging of hepatic hydatid disease, are not usually seen on USG or CT of skeletal muscle cysts, and calcification is rare. MRI is the examination of choice in case of suspicion of intramuscular hydatid disease. This is due to its ability to adequately demonstrate most features of hydatid disease, with the exception of calcifications. MRI images may identify various patterns of intramuscular hydatidosis such as its peripheral rim (also known as the rim sign), the membranes inside the cyst, peripheral oedema and peripheral enhancement with gadolinium which occurs due to the vascularization of the pericyst. In soft tissues, the mother cyst typically contains multiple smaller, secondary daughter cysts [14].

Based on typical imaging findings of a multilocular encapsulating cyst with multiple daughter cysts along with mild peripheral enhancement on post contrast study, a diagnosis of intramuscular hydatidosis was made preoperatively in our case.

It is crucial to identify or suspect the presence of a hydatid cyst before surgery, or else the cyst’s contents may be ruptured and may spill into the systemic circulation as a result of improper handling during surgical resection. This may induce an anaphylactic reaction as discussed previously [10,15]. The treatment of choice at present is complete surgical excision of the cyst along with thorough irrigation of the surrounding soft tissues with hypertonic saline to prevent recurrence. These procedures should be combined with the use of systemic antiparasitic drugs after surgery [8]. The patient must be kept under long-term clinical evaluation after surgery, to check for recurrence.

## 6 Conclusion

Primary intramuscular hydatidosis is an extremely rare manifestation of Hydatid disease which is endemic in India. The diagnosis of this disease proves challenging and requires a multimodal approach. The patient presented to us with a large swelling over his left thigh and a diagnosis was established with detailed history, clinical examination along with radioimaging. The patient was treated with pericystectomy for all the cysts along with evacuation of daughter cysts followed by albendazole therapy.

## Sources of funding

This research did not receive any specific grant from funding agencies in the public, commercial, or not-for-profit sectors.

## Ethical approval

No ethical clearance was needed for this case report.

## Consent

Written informed consent was obtained from the patient for publication of this case report and the accompanying images. A copy of the written consent is available for review by the Editor-in-Chief of this journal on request.

## Author contribution

Dr Vaibhav Lakhanpal: Writing the paper.

Dr Mohit Badgurjar: Operated, revised and edited manuscript.

Dr Pankaj Saxena: Operated.

## Registration of research studies

This was not a first in man study.

## Guarantor

Dr Mohit Badgurjar.

## Provenance and peer review

Not commissioned, externally peer-reviewed.

## Patient perspective

I am thankful to the team of surgeons for accurately detecting my problem, operating on it and treating me so I could rejoin my work with full vigour.

## Declaration of Competing Interest

The authors report no declarations of interest.
